# Assessment of Static Balance Metrics in Community-Dwelling Older Adults Categorized Using the Fall Risk Appraisal Matrix

**DOI:** 10.3390/ijerph22071079

**Published:** 2025-07-06

**Authors:** Jethro Raphael M. Suarez, Joon-Hyuk Park, Ladda Thiamwong

**Affiliations:** 1 Department of Mechanical and Aerospace Engineering, University of Central Florida, Orlando, FL 32816, USA; jethroraphael.suarez@ucf.edu; 2 College of Nursing, University of Central Florida, Orlando, FL 32826, USA; ladda.thiamwong@ucf.edu; 3 Department of Robotics and Mechatronics Engineering, Daegu Gyeongbuk Institute of Science and Technology, 333 Techno Jungang Daero, Hyeonpung-eup, Dahseong-gun, Daegu 42988, Republic of Korea; 4 Disability, Aging, and Technology Cluster, University of Central Florida, Orlando, FL 32816, USA

**Keywords:** BTrackS, sway area, fear of falling, directional sway

## Abstract

The Fall Risk Appraisal Matrix (FRAM) is a simple fall risk assessment tool that categorizes older adults into four separate groups based on their fear of falling (FOF) and static balance performance. Static balance for the FRAM is evaluated solely by postural sway distance, which does not account for other static balance parameters, such as sway area, anterior–posterior (AP) sway range, medial–lateral (ML) sway range, and sway velocity. The objective of this study was to compare these additional metrics across the FRAM groups to assess their relevance and validity for inclusion in static balance performance assessment. Hence, these measures were compared among the four different fall risk groups within the FRAM (203 participants; mean age = 75.0 ± 7.2 years) using Kruskal–Wallis test, followed by Dunn’s post hoc tests with Bonferroni correction. All balance metrics were significantly greater in the Incongruent (poor balance/low FOF) and Congruent (poor balance/high FOF) groups than the Rational (good balance/low FOF) group, as well as in the Congruent group than the Irrational (good balance/high FOF) group (*p* < 0.001). Additionally, AP sway range and sway velocity significantly differed between the Irrational and Incongruent groups (*p* < 0.001). The results suggest that the inclusion of these additional static balance measures, in addition to sway distance, reveals specific tendencies in static balance among different fall risk groups, which can serve as a reference for other researchers and future studies to develop more individually tailored intervention programs based on their static balance specificities.

## 1. Introduction

Fall risk assessment tools are essential for developing effective intervention programs geared towards preventing falls in older adults [1], and there are many tools such as physical function tests (e.g., Timed-Up-and-Go and Short Physical Performance Battery) and self-reported questionnaires (e.g., Stopping Elderly Accidents, Deaths, and Injuries questionnaire and the Austin Health Falls Risk Screening Tool) [2,3,4,5]. A simple and cost-effective fall risk assessment tool for older adults that considers static balance performance and level of fear of falling (FOF) is the Fall Risk Appraisal Matrix (FRAM), which consists of four quadrants/categories: Rational (good static balance and low FOF), Irrational (good static balance and high FOF), Incongruent (poor static balance and low FOF), and Congruent (poor balance and high FOF) [6]. The static balance metric utilized in the FRAM for categorization is the postural sway distance measured during a static balance test. Postural sway distance represents the total distance that an individual’s center-of-pressure (COP) travels while standing on a force plate, providing a simple representation of static balance performance [7]. Although this metric directly reflects one’s postural sway and resulting COP changes during standing, it is a purely distance-based measure that does not capture other sway-related characteristics, such as the area of sway, directions of sway, or speed of sway. For example, a high value of postural sway distance, without considering the overall sway area and speed, could denote either sway over a larger area at a slower sway speed or sway over a smaller area at a faster sway speed. Various factors have been found to cause high postural sway, such as weakness in hip and ankle muscles, loss of somatosensory input from the lower limbs due to peripheral neuropathy, and feelings of insecurity within certain limits of stability [8,9,10]. Still, the extent to which these conditions negatively affect an individual’s static balance performance would not be fully understood by examining postural sway distance alone. Consideration of other static balance metrics could help to better understand the specificities of an individual’s sway tendency and drive the creation of tailored static balance training methods.

The inclusion of other static balance metrics, such as sway area and the ranges of sway in the medial–lateral (ML) and anterior–posterior (AP) directions, has been explored in previous studies to gain a better understanding of static balance performance [9,10,11,12,13,14,15,16,17]. A previous study compared the static balance performance of young and older adults under eyes-open and eyes-closed conditions using a force plate and found that both young and older adults had significantly greater sway in the ML direction during the eyes-closed condition when compared to the eyes-open condition, but only the older adults had a considerably larger sway in the AP direction during the eyes-closed condition [15]. These differences would not have been recognized without considering metrics beyond sway distance alone. Additionally, regarding falls, previous work has found that ML sway and sway speed are among the indicators that show significant associations with future falls among older adults, specifically [18]. The consideration of a variety of static balance metrics enables a comprehensive understanding of static balance performance, focusing on specific areas that may require improvement to prevent adverse outcomes, such as falls. However, the association between metrics such as directional movement (ML and AP movement) and sway speed in community-dwelling older adults has not been explored; therefore, investigation is warranted.

The FRAM has been previously used to screen community-dwelling older adults for fall risk [6,19]. The four groups within the FRAM have previously been compared against one another regarding physical activity levels, but balance metrics beyond postural sway distance have not been considered [20]. It has been found that as sway distance increases, other static balance metrics such as sway area, sway velocity, and both AP and ML directional sway tend to increase as well [14]. It has also been found that individuals with higher levels of FOF performed worse on static balance assessments than those with minimal to no FOF [21]. However, it is unknown whether such trends are consistent between the groups within the FRAM, due to the lack of between-group comparisons of static balance metrics.

Due to the aforementioned limitations of balance assessment using only the postural sway distance, the objective of this study was to compare static balance metrics beyond postural sway distance, namely sway area, AP sway range, ML sway range, and sway velocity, between community-dwelling older adults within each of the four groups in the FRAM. This aims to gain a clearer understanding of the balance performance of each group and what can be further informed by these additional metrics. It was hypothesized that all balance metrics will increase—indicating decreased balance performance—as postural sway distance increases independent of FOF, due to previously found positive relationships between sway distance and other balance metrics, namely sway area, AP sway, ML sway, and sway velocity [14]. Furthermore, an increase in static balance metrics will be exhibited as a result of an increase in FOF alone, consistent with the literature [21]. Since increased postural sway distance and FOF have been found to contribute to reduced static balance performance individually, increases in both postural sway and FOF simultaneously will result in increased static balance metrics, indicating decreased balance performance.

## 2. Materials and Methods

### 2.1. Setting and Sample Size

The present study is a cross-sectional investigation that stems from a larger study, federally funded by the National Institute on Minority Health and Health Disparities (R01MD018025), and pre-registered on ClinicalTrials.gov (NCT05778604). The protocol for the study was approved by the University of Central Florida Institutional Review Board (STUDY00003206, approved on 8 September 2021), adhered to the Declaration of Helsinki, and has been previously published elsewhere [22]. The study took place in the greater Orlando, FL, USA, metropolitan area, and recruitment was accomplished through word-of-mouth, flyer distribution, and partnership with local communities. A total of 246 community-dwelling older adults were recruited. Participants were included in this study if they were at least 60 years of age, had a low-income status based on the 2019 United States Census poverty thresholds relative to family size [23], completed the Short Falls Efficacy Scale International (Short FES-I) questionnaire, and completed a balance assessment using the BTrackS™. Participants who were unable to stand unassisted for at least 2 min (the maximum duration of each static balance assessment) or who were receiving treatment at any form of rehabilitation facility were excluded. After screening for inclusion and exclusion criteria, 203 participants were included in this study. All participant data was deidentified and securely stored and managed using Research Electronic Data Capture (REDCap), a secure, web-based application designed to store data for research [24].

### 2.2. Balance Assessment

The Balance Tracking System (BTrackS™; Balance Tracking Systems Inc., San Diego, CA, USA) is a cost-effective force plate system that measures multiple static balance metrics and was used for static balance assessment. The static balance metrics utilized for this study were sway area (95% ELL; the area of the smallest ellipse fitting 95% of the COP path), AP sway range (RG-AP; the difference in the maximum and minimum COP value along the y axis), ML sway range (RG-ML; the difference in the maximum and minimum COP value along the x axis), and sway velocity (VEL; the total COP path length divided by trial duration). Static balance metrics were assessed using the BTrackS™ Balance and Fall Risk Protocol administered via the BTrackS™ Assess Balance software (Version 7.5.5, Balance Tracking Systems Inc., San Diego, CA, USA). All static balance assessments were administered at various senior centers across the greater Orlando area for the convenience of participants. Participants were instructed to remove their shoes or sandals and stand on the BTrackS™ force plate with their feet approximately 30 cm apart and facing forward (toe-out angle = 0°). Participants performed four 20 s trials with their hands on their hips, head facing forward, and eyes closed. The first trial served as a practice trial, whereas the following three trials were averaged to calculate the COP metrics. For respondent safety, a researcher placed a hand near the participant’s back to ensure that the participant did not fall at any point during the trials, and participants were given approximately 10 s of rest between trials. All procedures follow the directions given by the BTrackS™ Balance and Fall Risk Protocol. Participants were asked to refrain from consuming any form of caffeine on the day of the assessment. The COP values from the BTrackS™ were obtained by sampling the four load cells within the force plate every 40 milliseconds, then passing through a 2nd order, low-pass Butterworth filter with a 4 Hz cut-off frequency. An example of the results from the BTrackS™ Balance and Fall Risk Protocol is mentioned elsewhere [25]. The BTrackS Balance and Fall Risk Protocol has been found to have an excellent intraclass correlation coefficient (ICC = 0.83) and has been utilized in previous studies involving older adults [26,27].

### 2.3. Fear of Falling

The Short Falls Efficacy Scale International (Short FES-I) is a seven-item self-report questionnaire that assesses the level of Falls Efficacy of an individual, serving as a shorter version of the FES-I. The questions on the Short FES-I ask about the level of concern an individual has regarding falling in specific situations, such as going up and down stairs, taking a bath or shower, and getting in and out of a chair. Each question utilizes a 5-point Likert scale ranging from 0 (“Not at all concerned about falling”) to 4 (“Very concerned about falling”). The total score of the Short FES-I ranges from 7 to 28, with a total score of 7 to 8 representing a Low Fear of Falling, a score of 9 to 13 representing a Moderate Fear of Falling, and a score of 14 to 28 representing a High Fear of Falling. The Short FES-I has been found to exhibit excellent test–retest reliability (Cronbach’s alpha = 0.92, ICC = 0.83) and correlates well with the FES-I (0.97) [28]. For this study, participants completed the Short FES-I independently. A researcher was only involved during the completion of this questionnaire if a participant needed clarification regarding a prompt on the Short FES-I.

### 2.4. Fall Risk Appraisal Matrix (FRAM)

**Figure 1:**
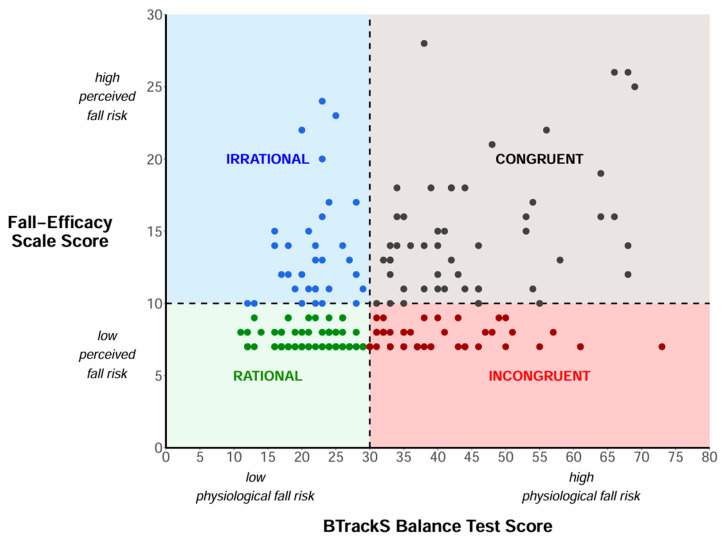
Distribution of 203 community-dwelling older adults within the FRAM. Notes. Rational group = good balance and low FOF; Irrational group = good balance and high FOF; Incongruent group = poor balance and low FOF; Congruent group = poor balance and high FOF.

The FRAM utilizes postural sway distance from the BTrackS™ and the level of FOF from the Short FES-I to categorize older adults into four categories: Rational (good balance and low FOF), Irrational (good balance and high FOF), Incongruent (poor balance and low FOF), and Congruent (poor balance and high FOF). Postural sway distance is plotted on the horizontal axis (x-axis), with values less than 30 cm indicating a low physiological fall risk (good balance) and values of 30 cm and higher indicating a high physiological fall risk (poor balance). Levels of FOF are plotted on the vertical axis (y-axis), with values below 10 indicating low psychological fall risk and values of 10 and above indicating high psychological fall risk. The FRAM can be created and edited in R software (Version 4.3.1, Posit, Boston, MA, USA) to easily include participant data (e.g., Figure 1) [29].

### 2.5. Statistical Analysis

All statistical analysis was performed using RStudio (Version 4.3.1, Posit, Boston, MA, USA). All balance metrics exhibited non-normality, as found through Anderson–Darling tests. As a result, Kruskal–Wallis test was used for comparative analysis between FRAM groups. Statistical significance was set at *p* < 0.05. Post hoc analysis was performed using Dunn’s test with the application of Bonferroni correction to account for multiple comparisons. The adjusted significance level for post hoc analysis after Bonferroni was set at *p* < 0.002. Effect sizes (r) were calculated using the total sample size and the Z-statistic output by each Dunn’s test.

## 3. Results

After screening, a total of 203 community-dwelling older adults were included in this study, with 68 (33%) in the Rational group, 32 (16%) in the Irrational group, 47 (23%) in the Incongruent group, and 56 (28%) in the Congruent group. The distribution of participants in the FRAM is illustrated in Figure 1, and participant demographics for each group are presented in Table 1.

**Figure 2:**
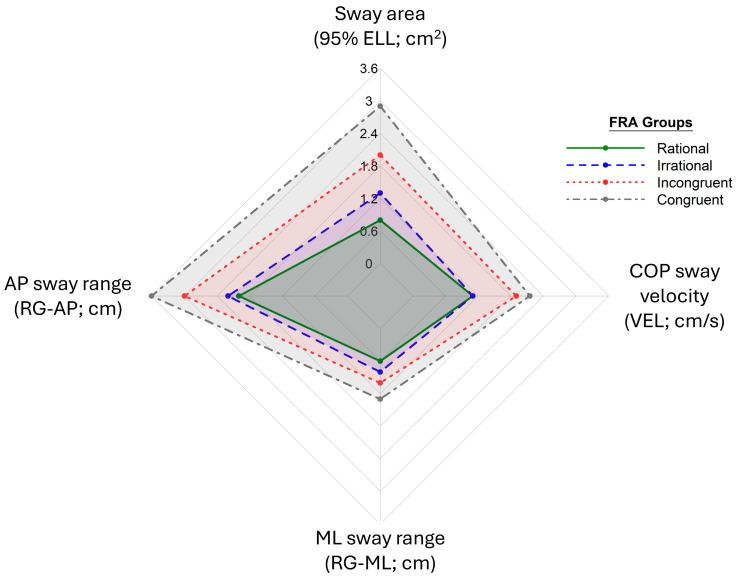
Spider chart of median values for sway area, AP sway range, ML sway range, and COP sway velocity for each FRAM group.

The Kruskal–Wallis test revealed significant differences in all four balance metrics among the four FRAM groups (*p* < 0.001). Median values and interquartile ranges (IQRs) for each balance metric in each FRAM group are presented in Table 2. In contrast, a visual comparison of the balance metrics among FRAM groups is presented in Figure 2. The results of the post hoc analysis, along with the percent differences between FRA groups for each static balance metric, are presented in Table 3. Through post hoc analysis using Dunn’s test, all balance metrics were found to be significantly greater in the Incongruent and Congruent groups compared to the Rational group, as well as the Congruent group compared to the Irrational group, after applying the Bonferroni correction. At least a 50% increase in all balance metric medians was found in these comparisons, with the highest difference being a 263% increase in sway area in the Congruent group compared to the Rational group. All other inter-group comparisons using these metrics yielded non-significant differences, except that RG-AP and VEL additionally showed significantly greater values in the Incongruent group compared to the Irrational group (a 36% and 73% increase, respectively; *p* < 0.001).

## 4. Discussion

In the presented study, sway area (95% ELL), AP sway range (RG-AP), ML sway range (RG-ML), and sway velocity (VEL) were found to be significantly greater in the Incongruent and Congruent groups when compared to the Rational group, as well as in the Congruent group when compared to the Irrational group. A 50% increase or more was observed in all balance metrics across these comparisons. Furthermore, AP sway range and sway velocity were significantly greater in the Incongruent group than the Irrational group (a 36% and 73% increase, respectively). These results partially support the original hypothesis, showing that all balance metrics increased as postural sway distance increased, independent of FOF levels, as well as both postural sway distance and FOF increased. However, contrary to the hypothesis, an increase in levels of FOF independent of postural sway distance did not statistically affect these static balance metrics.

It was found that as postural sway distance increased, sway area, ranges of sway in both the ML and AP direction, and sway velocity increased. While this was an expected result, given the positive relationship found between sway distance, sway area, AP sway, ML sway, and sway velocity in a previous study [14], the results from the present study provide quantitative support for this (Table 3). When postural sway distances were similar between groups, sway area, AP sway range, ML sway range, and sway velocity did not differ due to increased levels of FOF alone, as indicated by the insignificant differences between the Rational and Irrational groups, as well as between the Congruent and Incongruent groups. FOF has been previously found to negatively affect the balance of older adults, as evidenced by significantly lower scores on assessments such as the Berg Balance Scale and Tinetti Performance-Oriented Mobility Assessment [21]. However, when controlling for postural sway distance, increased levels of FOF did not significantly change sway area, AP sway range, ML sway range, and sway velocity.

The insignificant differences resulting from increased levels of FOF, independent of changes in postural sway distance, may be due to an increase in perception, awareness, or a feeling of instability alone, rather than physical instability itself. Older adults with higher self-reported levels of FOF may be more sensitive to movement during balance tests due to their FOF, resulting in a perception of poor balance performance that is worse than their objectively measured balance performance. It has been found that older adults who experience FOF also experience a form of muscle tension or contraction [30,31,32,33]. This type of muscle reaction is likely an autonomic response due to the fear associated with falling and could prevent fall events, as well as reduce significant postural sway, by providing increased stabilization during standing, depending on the stance performed during a static balance assessment. Previous work that simulated FOF through an elevated platform found that a decrease in the amplitudes of oscillation in the AP direction occurred when individuals were elevated on a platform due to a postural freezing phenomenon, which has been referred to be synonymous with immobility and muscle stiffness [34,35,36,37]. While this type of FOF may differ from self-reported FOF (such as the type presented in this study), the findings of these studies support the idea that the muscle contraction may explain the insignificant differences in balance metrics due to changes in FOF alone. On the contrary, previous work has found that adults with FOF exhibit increased COP displacement [38]. However, such work involved inducing FOF through an elevated platform, as well as an eyes-open condition, which may have caused the increased COP displacement due to depth perception at elevated levels or other visual-related factors. Regarding the present study, the stance utilized by the BTrackS™ Balance and Fall Risk Protocol may have naturally restricted movement due to a wide base of support and may have caused the insignificant differences in balance metrics due to FOF alone. There has been a reported instance where an older adult experienced muscle tension due to FOF while bent over and was unable to stay upright when standing [33]. The muscle contraction response may only be of concern when an individual is not already standing upright or in a position that has a narrow base of support. Regarding the results of this study, tension or muscle contraction may have occurred in older adults with FOF, but it did not significantly affect sway metrics, as the static balance assessment began with a fully upright stance with a wide base of support.

While the difference in balance metrics between the Rational and Irrational groups did not reach statistical significance, there were greater values in all balance metrics in the Irrational group compared to the Rational group, with medians increasing by up to 63% in all metrics except for sway velocity. As previously mentioned, the BTrackS™ assessment utilizes a single, feet-apart stance with a wide base of support, which naturally allows for easier postural sway in the AP direction than the ML direction. The higher values for each balance metric in the Irrational group, as well as inherently easier postural sway in the AP direction due to the BTrackS™ assessment method, could have contributed to the significant difference in AP sway range between the Irrational and Incongruent groups. Stances with a narrow base of support may result in increased ML sway. As for sway velocity, a significant increase was expected in the Incongruent group compared to the Irrational group, due to the positive relationship observed between postural sway distance and sway velocity [14]. Overall, the results of this study provide additional information on the static balance performance of older adults categorized by the FRAM, which may support the development of intervention programs tailored to improve balance and reduce fall risk of older adults in each FRAM group.

### Limitations and Future Directions

The strength of this study lies in its investigation of balance metrics beyond postural sway distance, comparing the FRAM groups to provide a clearer picture of balance performance between each group. This is the first study to reveal that FRAM groups with similar values of postural sway distance (Rational–Irrational and Incongruent–Congruent groups) do not statistically differ in sway area, AP sway range, ML sway range and sway velocity, regardless of increased levels of FOF. However, this study had some limitations.

Although the total sample size was large, there was an uneven distribution of sex (176 out of 203 participants were female), as well as participants across the FRAM groups, with the smallest sample size being in the Irrational group (N = 32). These uneven distributions could have contributed to the insignificant values found during post hoc analysis, as well as reduced the overall statistical power of the analyses. To our knowledge, this is the first study to compare static balance metrics between the groups within the FRAM and, therefore, is considered an exploratory analysis to identify any potential trends. Due to this, confounding factors, such as cognitive function or impairment, age, and medical conditions or histories, were not considered in this analysis. Additionally, while increased perceptions of balance are a possibility, reports of self-perceptions of balance were not collected from the older adults. These factors may also have affected the results of this study.

This study closely followed the directions of the Balance and Fall Risk Protocol outlined by the BTrackS™, which only includes an eyes-closed balance condition. However, this may be particularly challenging for individuals with visuo-spatial difficulties. Studies that assess static balance using methods other than the BTrackS™ include both eyes-closed and eyes-open conditions during static standing [38,39]. An eyes-open condition may provide additional information regarding an individual’s static balance performance; therefore, such a condition should be included in future work, despite the protocol requiring only an eyes-closed condition. Although all static balance assessments were administered using the same protocol, the environment in which the assessments took place was not always consistent. For the convenience of the participants, the static balance assessments were administered at various senior centers in the greater Orlando area. Due to the varying available space at each senior center, environmental conditions such as noise could not be controlled. Additionally, although caffeine intake was restricted before taking the static balance assessment, medication use was not controlled for.

While the FRAM was originally developed as a cost-effective fall risk assessment tool for community-dwelling older adults, its use is not limited to this population. Older adults of any income category can use the FRAM. The inclusion of only community-dwelling older adults in this study may limit the generalizability of these results to community-dwelling older adults. Most of the comparisons were found to be significant under a conventional statistical threshold of *p* < 0.05, but the multiple comparisons between groups required statistical correction, resulting in a decrease in the statistical threshold (*p* < 0.003), leading to insignificant comparisons. Additionally, the cross-sectional nature of this study limits the ability to draw causal inferences; therefore, the findings should be interpreted as associative rather than causal. Longitudinal or experimental studies are warranted to fully determine causality. Future work should aim to achieve close-to-equal sample sizes of sex and older adults in each of the FRAM groups, consider factors that could differ between groups (e.g., sex, BMI, past medical history), include older adults of other income categories, control for factors such as environmental noise and medication use, consider including an eyes-open balance condition, and include self-reported perceptions of balance from older adults.

## 5. Conclusions

The presented study examined sway area, AP sway range, ML sway range, and sway velocity between groups within the FRAM. Postural sway distance, the sole balance metric considered in the FRAM, exhibits a level of ambiguity due to the absence of knowledge regarding the direction, area, and velocity of sway. This level of ambiguity warranted investigation of balance metrics beyond postural sway distance. It was found that all balance metrics increased with higher levels of postural sway distance, as well as postural sway distance and FOF together, but balance metrics did not increase by FOF alone, possibly due to muscle contractions/stiffness because of FOF and the stance performed during the static balance assessment, which exhibited a wide base of support. Groups with similar ranges of postural sway distance are not guaranteed to have similar sway area and directional sway ranges. However, that was the case in this study. These results highlight the need to better understand the balance performance of older adults within each FRAM group, enabling the recognition of postural control mechanisms or musculoskeletal weaknesses that older adults may exhibit within a specific group. Decreasing FOF alone may not be sufficient for improving the static balance of older adults and preventing falls. Interventions should aim to decrease both FOF and postural static sway distance to minimize fall risk and ensure improved static balance performance. Additionally, considering multiple static balance metrics beyond postural sway distance alone is essential to gain a clear understanding of an individual’s static balance performance.

## Figures and Tables

**Table 1:** Participant demographics for each FRAM group.

Category	Variables	Rational (N = 68)	Irrational (N = 32)	Incongruent (N = 47)	Congruent (N = 56)	Total (N = 203)
Sex	Female	62	31	35	48	176
	Male	6	1	12	8	27
Age (years)	mean ± SD	73.4 ± 6.5	75.1 ± 8.1	75.4 ± 7.8	76.4 ± 7.0	75.0 ± 7.2
	Range	62–89	61–87	61–96	62–92	61–96
BMI (kg/m^2^)	mean ± SD	28.4 ± 5.4	30.5 ± 8.3	30.7 ± 6.2	31.7 ± 6.0	30.2 ± 6.4
General Health	Poor	0	3	0	0	3
	Fair	3	8	6	17	34
	Good	34	15	22	28	99
	Very good	28	6	14	11	59
	Excellent	3	0	5	0	8
Education	Lower than high school	9	7	7	6	29
	High school	37	12	23	25	97
	College or above	22	13	17	25	77
Financial Status	Much less than adequate	8	4	1	3	16
	Less than adequate	10	9	8	9	36
	Just enough	38	16	30	35	119
	More than enough	9	3	7	8	27
	Much more than enough	3	0	1	1	5
Living Status	Alone	43	18	26	34	121
	With partner/spouse	17	7	10	8	42
	With family/friends	8	6	11	10	35
	Other	0	1	0	4	5

**Table 2:** Median, IQRs, and distribution of sway area, AP sway range, ML sway range, and COP sway velocity for each FRAM group.

Balance Variable	Rational		Irrational		Incongruent		Congruent	
	Median (IQR)	*p*-Value	Median (IQR)	*p*-Value	Median (IQR)	*p*-Value	Median (IQR)	*p*-Value
Sway area (95% ELL; cm^2^)	0.8 (0.8)	<0.001 *	1.3 (1.0)	0.003 *	2.0 (1.4)	<0.001 *	2.9 (4.5)	<0.001 *
AP sway range (RG-AP; cm)	2.0 (0.9)	<0.001 *	2.2 (0.6)	0.054	3.0 (1.3)	<0.001 *	3.6 (2.1)	<0.001 *
ML sway range (RG-ML; cm)	0.6 (0.3)	<0.001 *	0.8 (0.4)	<0.001 *	1.0 (0.5)	<0.001 *	1.3 (1.2)	<0.001 *
COP sway velocity (VEL; cm/s)	1.1 (0.4)	<0.001 *	1.1 (0.2)	<0.001 *	1.9 (0.8)	<0.001 *	2.2 (0.8)	<0.001 *

* Non-normal distribution, as indicated by Anderson–Darling tests (*p*-value < 0.05).

**Table 3:** Results of post hoc analysis through Dunn’s test (*p*-values) and percent differences for sway area, AP sway range, ML sway range, and COP sway velocity medians between FRAM groups.

Group Comparison	Variable	Sway Area (95% ELL; cm^2^)	AP Sway Range (RG-AP; cm)	ML Sway Range (RG-ML; cm)	COP Sway Velocity (VEL; cm/s)
Rational– Irrational	Percent change (%)	+63%	+10%	+33%	+0%
	*p*-Value	0.067	1.000	0.111	1.000
	Effect size (r)	0.178	0.062	0.165	0.037
Rational– Incongruent	Percent change (%)	+150%	+50%	+66%	+73%
	*p*-Value	<0.001 *	<0.001 *	<0.001 *	<0.001 *
	Effect size (r)	0.432	0.424	0.407	0.583
Rational– Congruent	Percent change (%)	+263%	+80%	+77%	+100%
	*p*-Value	<0.001 *	<0.001 *	<0.001 *	<0.001 *
	Effect size (r)	0.641	0.574	0.551	0.706
Irrational– Incongruent	Percent change (%)	+35%	+36%	+25%	+73%
	*p*-Value	0.038	<0.001 *	0.056	<0.001 *
	Effect size (r)	0.191	0.293	0.182	0.448
Irrational– Congruent	Percent change (%)	+123%	+64%	+63%	+100%
	*p*-Value	<0.001 *	<0.001 *	<0.001 *	<0.001 *
	Effect size (r)	0.350	0.407	0.289	0.539
Incongruent– Congruent	Percent change (%)	+45%	+20%	+30%	+16%
	*p*-Value	0.091	0.582	0.655	1.000
	Effect size (r)	0.170	0.116	0.112	0.085

* Significant *p*-values after Bonferroni correction < 0.002.

## Data Availability

The data presented in this study are available on request from the corresponding author due to privacy restrictions.

## Abbreviations

The following abbreviations are used in this manuscript:

FRAM	Fall Risk Appraisal Matrix
FOF	Fear of falling
COP	Center of pressure
ML	Medial–lateral
AP	Anterior–posterior
95% ELL	95% elliptical sway area
RG-AP	Anterior–posterior sway range
RG-ML	Medial–lateral sway range
VEL	COP sway velocity
